# Intervention modalities for brain fog caused by long-COVID: systematic review of the literature

**DOI:** 10.1007/s10072-024-07566-w

**Published:** 2024-05-02

**Authors:** Alon Gorenshtein, Tom Liba, Liron Leibovitch, Shai Stern, Yael Stern

**Affiliations:** 1 Azrieli Faculty of Medicine, Bar-Ilan University, Safed, Israel; 2 Maccabi Healthcare Services, Tel Aviv-Yafo, Israel

**Keywords:** Post-acute sequelae of COVID-19, Brain fog, Intervention, Oxygen therapy hyperbaric, Non-invasive brain stimulation

## Abstract

Individuals suffering from long-COVID can present with “brain fog”, which is characterized by a range of cognitive impairments, such as confusion, short-term memory loss, and difficulty concentrating. To date, several potential interventions for brain fog have been considered. Notably, no systematic review has comprehensively discussed the impact of each intervention type on brain fog symptoms. We included studies on adult (aged > 18 years) individuals with proven long- COVID brain-fog symptoms from PubMed, MEDLINE, Central, Scopus, and Embase. A search limit was set for articles published between 01/2020 and 31/12/2023. We excluded studies lacking an objective assessment of brain fog symptoms and patients with preexisting neurological diseases that affected cognition before COVID-19 infection. This review provided relevant information from 17 studies. The rehabilitation studies utilized diverse approaches, leading to a range of outcomes in terms of the effectiveness of the interventions. Six studies described noninvasive brain stimulation, and all showed improvement in cognitive ability. Three studies described hyperbaric oxygen therapy, all of which showed improvements in cognitive assessment tests and brain perfusion. Two studies showed that the use of Palmitoylethanolamide and Luteolin (PEA-LUT) improved cognitive impairment. Noninvasive brain stimulation and hyperbaric oxygen therapy showed promising results in the treatment of brain fog symptoms caused by long-COVID, with improved perfusion and cortical excitability. Furthermore, both rehabilitation strategies and PEA-LUT administration have been associated with improvements in symptoms of brain fog. Future studies should explore combinations of interventions and include longer follow-up periods to assess the long-term effects of these treatments.

## Introduction & background

More than 774,075,242 coronavirus disease 2019 (COVID-19) cases have been reported by the World Health Organization (WHO). As the world transitions into a postpandemic era, COVID-19 continues to persist in the form of various variants and subvariants [1]. Approximately 10–35% of COVID-19 survivors experience persistent symptoms such as fatigue, dyspnea, chest pain, cough, depression, anxiety, posttraumatic stress disorder, memory loss, and difficulty concentrating [2]. The National Institute for Health and Care Excellence (NICE) guidelines characterize the persistence of symptoms as long-COVID. According to these guidelines, long-COVID involves symptoms that persist for > 12 weeks (3 months) and cannot be attributed to an alternative diagnosis [3]. Moreover, one of the three patients with COVID-19 will be diagnosed with neurological symptoms within 6 months of infection [4]. Neurological symptoms characterized by impaired intellectual functions in individuals with long-COVID are collectively referred to as "brain fog," which encompasses a range of cognitive impairments, such as confusion, short-term memory loss, and difficulty concentrating [5, 6]. The mechanism underlying how long-COVID causes brain fog symptoms is not entirely understood. However, evidence indicates that COVID-19 may invade the brain through various possible routes. This invasion triggers neuroinflammatory processes that can activate cells such as astrocytes and microglia. These processes may contribute to the neurological symptoms observed in long-COVID patients [6]. Regarding the factors influencing brain fog caused by long-COVID, studies have shown that female patients and those who experienced a milder course of acute COVID tend to be more susceptible to developing brain fog [7].

The significance of cognitive function cannot be overstated because it plays a crucial role in our daily lives. Any impairment in cognitive function can have a severe impact on quality of life. Studies indicate that individuals experiencing brain fog often suffer from decreased occupational function, making it challenging for them to resume their normal occupations [8–10]. Furthermore, brain fog has been linked to depression and poor sleep quality [11, 12]. Given the adverse impact on quality of life and the widespread occurrence of brain fog, exploring interventions to improve quality of life and providing treatment are important areas of research.

To date, several potential interventions for brain fog have been considered, including noninvasive brain stimulation, hyperbaric oxygen therapy, and traditional and nontraditional rehabilitation approaches. Notably, no systematic review has comprehensively discussed the impact of each intervention type on brain fog symptoms. Therefore, the goal of this systematic review was to explore the effects of different intervention types on brain fog symptoms in those suffering from long-Covid.

## Methods

This study used the following methodological framework in conjunction with the extended Preferred Reporting Items for Systematic Reviews and Meta-Analyses (PRISMA) checklist for systemic reviews [13]. The study protocol was preregistered on the International Prospective Register Reviews (PROSPERO; CRD42024502977). The primary aim of this study was to describe interventions for brain fog caused by long-COVID. Due to the innovative nature of this review, the included studies applied diverse methodologies for documenting improvements and diagnosing brain fog symptoms, employing various tests to detect mild cognitive impairments. Additionally, considering the range of papers providing quantitative outcomes, conducting a meta-analysis might not be feasible. Therefore, narrative synthesis was the most appropriate for the different types of studies we found.

### Search strategy and selection criteria

This systematic review will encompass studies focusing on individuals who have exhibited confirmed brain fog symptoms attributed to long-COVID. from PubMed, Central, Scopus and Web of Science to establish an extensive pool of helpful information regarding brain fog symptoms. We intentionally set a search limit for the period from January 1, 2020, to December 31, 2023. This time frame was chosen because COVID-19 cases began to emerge in December 2019, marking the onset of the pandemic. The following search terms were used: “Post-Acute COVID-19 Syndrome” OR "COVID-19″ AND "Mental Fatigue,” OR "Cognitive Dysfunction,” OR "therapy,” OR "Cognitive Training,” OR "Hyperbaric Oxygenation,” OR "Transcranial Magnetic Stimulation”.

### Inclusion and exclusion criteria

We included studies with adult populations (≥ 18 years) exhibiting brain fog symptoms at least four weeks post-COVID-19 infection. Studies were required to provide specific descriptions of brain fog symptoms rather than relying on broad terminology such as 'cognitive impairment'. Furthermore, additional studies are needed to detail interventions aimed at addressing brain fog symptoms. We excluded studies lacking objective assessments of brain fog, those with unclear diagnoses, and those involving patients with preexisting neurological conditions affecting cognition. Additionally, we excluded non-English studies, systematic reviews, and meta-analyses.

### Data extraction and quality assessment

Screening and data extraction were performed by four independent reviewers (A.G., T.L., S.S., and L.L.). Any disagreements were discussed, and a consensus was reached by four reviewers. For studies that reported on the control and patient groups, only patient data were extracted and used, as per the decision to combine both clinical trials and observational studies. The following data were retrieved for each article: first author's name, location, publication time, study type, number of long COVID-19 patients, outcome measure, intervention modality, duration of treatment, number of sessions, adverse effects, primary study findings, and secondary outcomes. Four authors (A.G., T.L., S.S., and L.L.) independently extracted information from the full texts of the 16 selected studies. Inconsistencies between the reviewers were resolved through consultation with a senior reviewer (Y.S.).

For quality assessment, two authors (A.G. and T.L.) independently assessed (1) the criteria for the diagnosis of COVID-19, (2) the duration of the intervention methods used for assessing brain-fog symptoms caused by long-COVID, and (3) the scoring system used to assess brain-fog symptoms.

### Risk of bias assessment

**Fig. 1:**
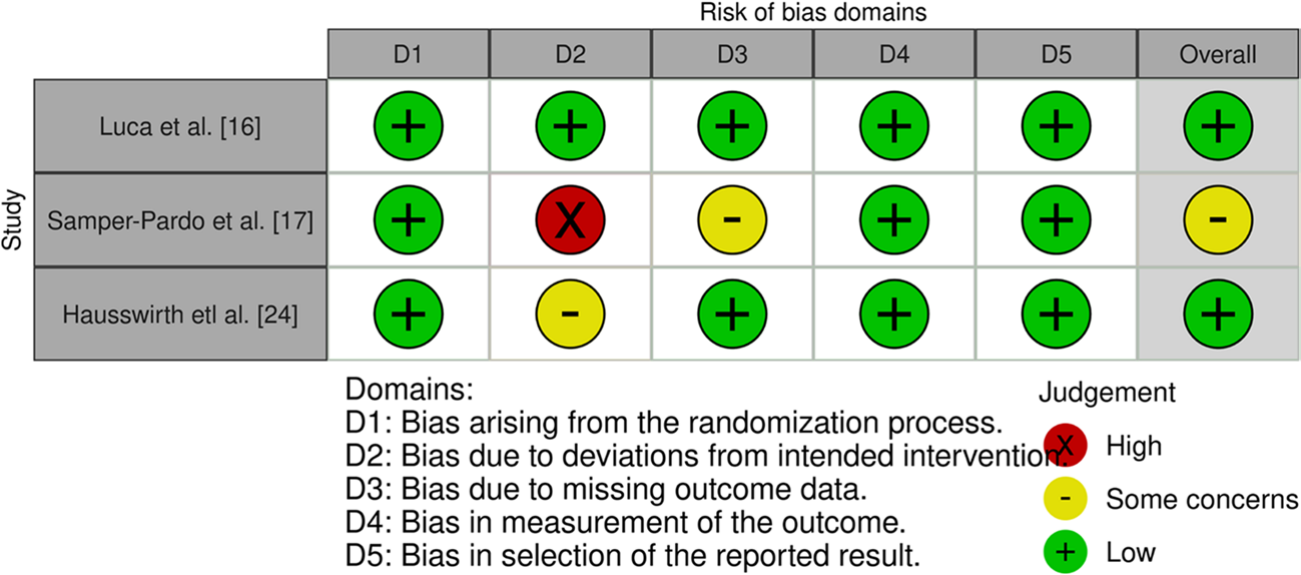
Revised Cochrane risk-of-bias (ROB2) tool for RCTs

Risk of bias assessment for cohort studies was performed using the Newcastle–Ottawa Scale (NOS) (Table 1), and for randomized clinical trials (RCTs), risk of bias was assessed using the revised Cochrane risk-of-bias (ROB2) tool for RCTs (Fig. 1). In evaluating the included case reports, case series, and pilot studies, a comprehensive approach was used to assess the quality and reliability of the evidence presented. Each case report was scrutinized for clarity of reporting, objectivity of information, adherence to diagnostic criteria, appropriateness of treatment interventions, and transparency in outcome measures. Special attention was given to alternative explanations for the observed clinical findings, ethical considerations such as patient consent and confidentiality, and disclosures of conflicts of interest. Furthermore, the generalizability of the reported cases to broader clinical practice was considered, along with the educational value they provided. For pilot studies, particular emphasis was placed on methodological rigor, including clarity of research objectives, appropriateness of study design, transparency in data collection and analysis, and consideration of potential biases. Overall, this comprehensive approach enabled a thorough assessment of the strengths and limitations of the individual case reports, case series, and pilot studies included in the review, contributing to a nuanced understanding of the evidence base for the evaluated interventions and clinical phenomena.

**Table 1:** Risk of bias quality assessment of cohort studies using the Newcastle–Ottawa Scale

				Selection			Comparability		Exposure/Outcome			Sub total assessment		Conclusion	Total
Studies	Study type	1	2	3	4	1a	1b	1	2	3	S	C	E	Column	Column
Kupferschmitt et al. [15]	cohort	*	*	*	*	*	*	*	no	*	good	good	good	good	8
Sasaki et al. [16]	cohort	*	*	*	*	*	no	*	no	*	good	fair	good	good	7
Rabaiotti et al. [19]	cohort	*	*	*	*	*	*	*	no	*	good	good	good	good	8
Braga et al. [22]	cohort	*	*	*	*	*	*	*	*	*	good	good	good	good	9
Tim Robbins et al. [29]	cohort	*	*	*	*	*	no	*	no	*	good	fair	good	good	7
Cenacchi et al. [30]	case–control	*	*	*	*	*	*	*	*	*	good	good	good	good	9

asterisk (*) signify one star based on the star system of Newcastle-Ottawa Scale

## Results

**Fig. 2:**
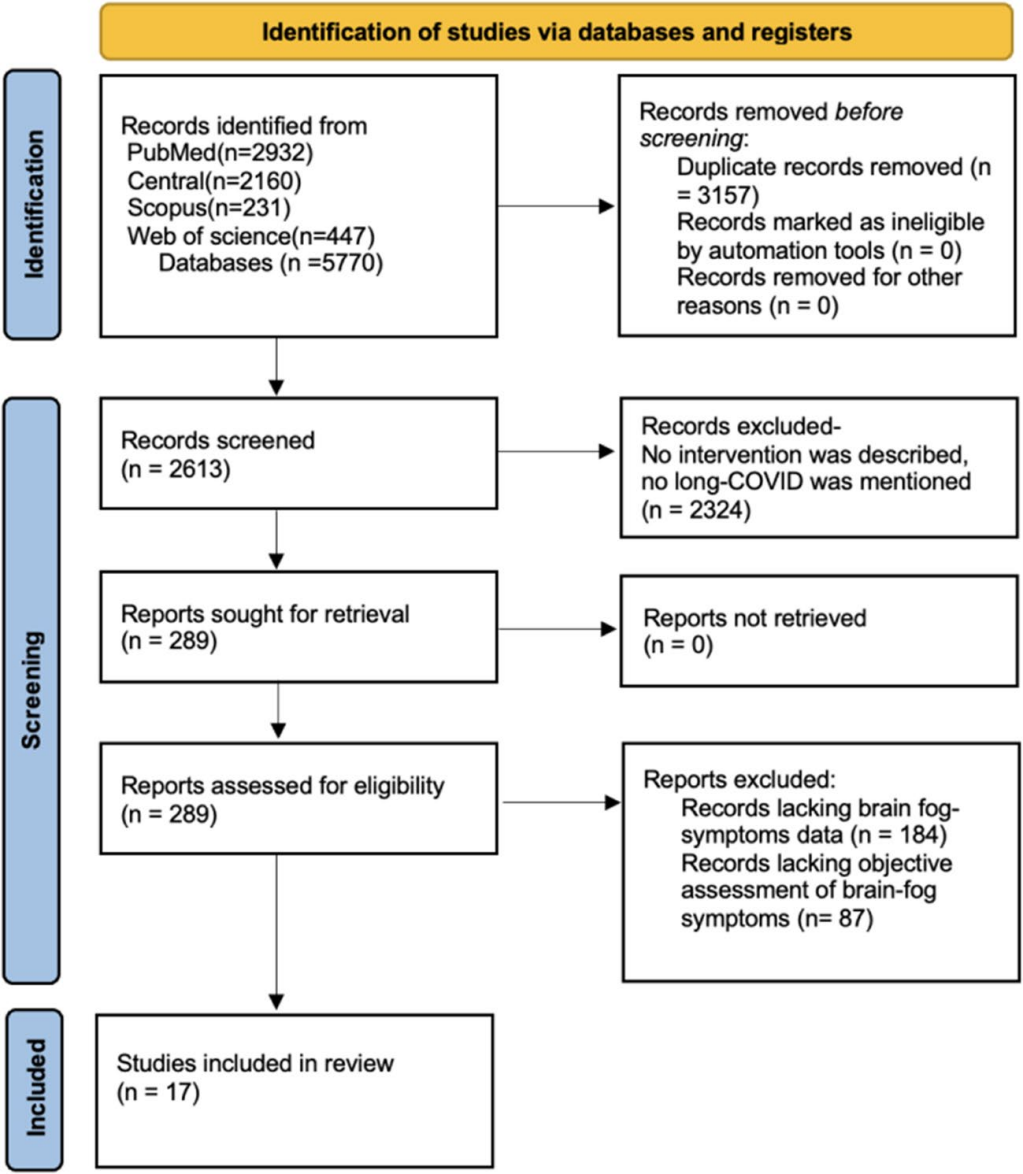
Preferred reporting items for systematic reviews and meta-analyses (PRISMA) Flow Chart

In total, 5770 articles were reviewed after the removal of duplicates, 2613 articles were screened for titles and abstracts, and out of them, 287 articles met the criteria. These studies provided information on possible interventions to treat neurocognitive deficits or long-COVID. Subsequently, the full texts of these articles were evaluated following the inclusion and exclusion criteria that were issued above; after quality assessment, 17 studies were included in this review [14–30]. Figure 2 shows the flow diagram detailing the review process and study selection based on the PRISMA flow chart.

### Characteristics of the results

Table 2 presents the characteristics and findings of the included studies. The sample sizes ranged from one to 208. The earliest publication date was 01/11/2021, while the latest was 20/03/2024. Thirteen countries were included in the review: the USA, Norway, the United Kingdom, Spain, Italy, Germany, Taiwan, Israel, Poland, the United Arab Emirates, Brazil, France, and Japan. The following study types were included: cohort, observational, exploratory, pilot study, clinical trial, case report, and case series. A total of 806 patients were diagnosed with COVID-19. A variety of assessment tools were employed to evaluate brain fog, including the Wechsler Memory Scale (WMS), Third Edition, Montreal Cognitive Assessment (MoCA), Test Battery for Attention (TAP), Auditory Verbal Learning Test (AVLT), Wechsler Adult Intelligence Scale (WAIS4)-Fourth Edition, Mini-Mental State Examination (MMSE), NeuroTrax computerized testing battery, CANTAB cognitive research software, Barrow Neurological Institute Screen for Higher Cerebral Functions (BNIS), Cognitive Assessment Battery (CAB), Psychology Experiment Building Language (PEBL), Prospective–Retrospective Memory Questionnaire (PRMQ), Fatigue Severity Scale (FSS), and Perceived Deficits Questionnaire-Depression 5-item (PDQ-D-5).

Several intervention modalities, such as rehabilitation (individualized psychological intervention of cognitive and cognitive behavioral therapy, personalized computerized cognitive training, aerobic exercise training, body awareness training, breathing therapy, mobile application, and mindfulness-based interventions), noninvasive brain stimulation (transcranial magnetic stimulation, theta burst stimulation, transcranial direct current stimulation, transcranial alternating current stimulation, and photobiomodulation), hyperbaric oxygen therapy and pharmacological therapy (palmitoylethanolamide and luteolin (PEA-LUT)), have been studied (Table 2).

**Table 2:** Characteristics of the included studies

References	Country	Published time	Study type	No. of patient with post-Covid-19	Outcome measure	Intervention modality	Study findings
Kupferschmitt et al. [15]	Germany	31/10/2023	Cohort	80	MoCa + TAP	Rehabilitation(multi-modal-Cognitive behavioral therapy,TAP, cognitive training in a group setting and indivudal,aerobic exercise training, body awareness training, breathing therapy,)	Cognitive impairments persist throughout rehabilitation despite treatment
Sasaki et al. [16]	Japan	28/02/2023	Cohort	12	WAIS4	Noninvasive brain stimulation (transcranial Magnetic StimulationS)	All WAIS4 sub-items were significantly improved after repetitive transcranial magnetic stimulation intervention. Hypoperfusion in the bilateral occipital and frontal lobes observed on SPECT improved in extent and severity after ten sessions of repetitive transcranial Magnetic Stimulation
Luca et al. [17]	Italy	17/08/2022	Clinical trial	69	MMSE	PEA-LUT,olfactory training(three groups- 1-NAÏVE-1, PEA-LUT plus olfactory training 2-NAÏVE-2, PEA-LUT alone 3-Individuals previously exposed to olfactory training = reciceve PEA-LUT and contiue olfactory training)	Mental clouding showed a statistical reduction in severity between the baseline and three months after treatment for group 1 and 3. Group 2 did not show significant reduction
Samper-Pardo et al. [18]	Spain	16/05/2023	Clinical trial	100	MoCA	Telerehabilitation( phone application with contents such as-Recommendations, Cognitive stimulation exercises, Participation in community resources and respiratory physiotherapy exercises.)	After the intervention, no significant differences were found in favour of the group intervention.MoCa scores increased. In both control and intervention group
Rabaiotti et al. [19]	Italy	12/05/2023	Clinical trial	64	MoCA	Rehabilitation (individualized psychological intervention of cognitive stimulation on top of a standard in-hospital rehabilitation program)	The mean MoCA overall was higher in discharge compare to before admisson, corresponding to a significant improvement in cognitive function between admission and discharge (20.4 ± 5 vs. 24.7 ± 3.7)
M. Bhaiyat et al. [20]	United Arab Emirates	15/02/2022	Case-report	1	NeuroTrax computerized testing battery	Oxygen therapy hyperbaric	The post-hyperbaric oxygen therapy neurocognitive testing showed significant improvement in global memory with the most dominant effect being on nonverbal memory, executive functions, attention, information procession speed, cognitive flexibility, and multitasking. significant improvements in brain perfusion and microstructure by magnetic resonance imaging
Wysokiński et al. [21]	Poland	12/08/2023	Case-report	1	CANTAB	Noninvasive brain stimulation (transcranial direct current stimulation) + cognitive rehabilitation	Visual memory scores improved at visit 2 and returned to the baseline level at visit 3. Mixed results were observed for multitasking, emotion recognition, response speed, attention. No changes or transient decrease at visit 2 were seen for working memory
Braga et al. [22]	Brazil	07/11/2023	Cohort	208	BNIS	Rehabilitation	Several statistically significant gains on the BNIS (overall score, attention/concentration subscale, visuospatial problem-solving scale) compared to the first formal neuropsychological evaluation. The percentage of patients with results below the BNIS Total z-score cut-off (z score < –1) fell from 54% in the first evaluation to 33% at follow-up
Sabel et al. [23]	Germany	28/12/2021	Case-report	2	TAP + AVLT*	Noninvasive brain stimulation (transcranial alternating current stimulation)	In both patients NIBS markedly improved cognition. Cognitive tests in one patient confirmed recovery of up to 40–60% in cognitive subfunctions
Duñabeitia et al. [24]	Norway	10/02/2023	Pilot study	73	CAB	Rehabilitation (personalized computerized cognitive training)	There was a consistent increase in the scores obtained in the cognitive assessment after the CCT (i.e., at post-test) as compared with those at baseline, and this increase extended to the five measured cognitive domains
Hausswirth etl al. [25]	France	11/01/2023	Clinical trial	34	PEBL	Mindfulness-based interventions	Neuro-meditation using the Rebalance® device has significantly improve mood, physical, and mental fatigue, as well as cognitive functioning in the intervention group compare to control (i.e., no evident cognitive improvement of the control group)
Noda et al. [26]	Japan	28/12/2022	Case-series	23	PDQ-D-5	Noninvasive brain stimulation(repetitive transcranial magnetic stimulation + intermittent theta burst stimulation)	The cognitive function assessed by PDQ-D-5 improved from 10.0 (± 5.2) to 6.3 (± 4.7), showing a significant improvement in cognitive function, with none of the patients showing deterioration in their cognitive function, following the TMS treatment
Zilberman-Itskovich et al. [27]	Israel	12/07/2022	Clinical Trial	73	NeuroTrax computerized testing battery	Oxygen therapy hyperbaric	Following HBOT, there was a significant group-by-time interaction in global cognitive function, attention and executive function. Clinical outcomes were associated with significant improvement in brain MRI perfusion and microstructural changes in the supramarginal gyrus, left supplementary motor area, right insula, left frontal precentral gyrus, right middle frontal gyrus, and superior corona radiate
Bowen et al. [28]	USA	05/04/2023	Pilot study	14	MoCA	Noninvasive brain stimulation (Photobiomodulation)	Noted significant improvements in MoCA scores following tPBM
Robbins et al. [29]	United Kingdom	21/11/2021	Cohort	10	NeuroTrax computerized testing battery	Oxygen therapy hyperbaric	Hyperbaric oxygen therapy yielded a statistically significant improvement in the global cognition, executive function,attention,information processing and verbal function
Cenacchi et al. [30]	Italy	20/03/2024	Exploratory study	41	MoCA + PRMQ + FSS	PEA-LUT	Twenty-six patients treated with co-ultraPEALut showed a significant improvement in PRMQ (T0: 51.94 ± 10.55, T1: 39.67 ± 13.02, *p* < 0.00001) and MoCA raw score (T0: 25.76 ± 2.3, T1: 27.2 ± 2, p 0.0260); Patients treated with co-ultraPEALut and corticosteroids were not statistically different from those treated with co-ultraPEALut alone. Neuro-post-COVID-19 patients treated with co-ultraPEALut scored better than controls in MoCA and PRMQ questionnaires after 10 months:

Abbreviations: *WMS* Wechsler Memory Scale; *MoCa* Montreal Cognitive Assessment; *TAP* Test Battery for Attention); *AVLT* Auditory Verbal Learning Test; *WAIS4* Wechsler Adult Intelligence Scale; *MMSE* Mini-Mental State Examination; *BNIS* Barrow Neurological Institute Screen for Higher Cerebral Functions; *CAB* Cognitive Assessment Battery, *PEBL* Psychology Experiment Building Language; *PQD-D-5* Perceived Deficits Questionnaire-Depression 5-item; *PEA-LUT* Palmitoylethanolamide and Luteolin, *PRMQ* Prospective–Retrospective Memory Questionnaire; *FSS* Fatigue Severity Scale, out of the two patients, one underwent a cognitive test battery while the second one completed a self-report evaluation

### Duration of treatment

In this review, we examined the median time for treatment and the number of sessions required for each type of intervention. Our analysis revealed notable variations in both aspects across interventions. In terms of the median time for treatment, noninvasive brain stimulation emerged as the most expedient, demonstrating a median treatment duration of 13.5 days. This was followed by rehabilitation, which exhibited a median treatment duration of 32.5 days. Moreover, the median duration of hyperbaric oxygen therapy treatment was 56 days, while pharmacological intervention necessitated the longest median duration of treatment, with a duration of 75 days. Moreover, when examining the number of sessions needed, we noticed different patterns across interventions. Noninvasive brain stimulation required the fewest sessions, with a median of 12 sessions, while hyperbaric oxygen therapy required a median of 40 sessions. Table 3 summarizes the median and mean values for the number of sessions and treatment duration associated with each intervention for brain fog induced by long-COVID.

**Table 3:** Median and mean for the number of sessions and treatment duration for each intervention for brain fog induced by long-COVID

Intervention	Median duration	Mean duration	Median number of sessions	Mean number of sessions
Noninvasive brain stimulation	13.5	14.58 ± 8.4	12	12.75 ± 3.67
Rehabilitation	32.5	44.5 ± 24.68	-	-
Hyperbaric oxygen therapy	56	51.66 ± 37.68	40	36.66 ± 25.16
Pharmacological	75	75	-	-

### Adverse effects

Regarding adverse effects, noninvasive brain stimulation and hyperbaric oxygen therapy revealed mild adverse effects. The case report by Chang et al. presented a 30-year-old female with persistent anxiety, depression, insomnia, and brain fog symptoms for eight weeks after COVID-19 infection. Chang et al. used accelerated theta burst stimulation of the bilateral dorsolateral prefrontal cortex using an Apollo transcranial magnetic stimulation therapy system as an intervention. The adverse effects of the intervention were dizziness and headache; however, they were transient and resolved after treatment [14]. Noda et al. conducted a case series of 23 patients with long-term COVID-19. The intervention protocol consisted of one session of intermittent theta burst stimulation for the dorsolateral prefrontal cortex and one session of low-frequency repetitive transcranial magnetic stimulation for the right lateral orbitofrontal cortex with one transcranial magnetic stimulation treatment per day [26]. The adverse effect of the intervention was scalp pain at the stimulation site, which was reported by 4 out of 23 patients. In a clinical trial conducted by Zilberman-Itskovich et al., 73 patients with long COVID-19 were identified, with 37 and 36 patients in the intervention and control groups, respectively. The intervention group received 40 daily sessions of hyperbaric oxygen therapy. The following adverse effects were reported: barotrauma (*n* = 4), ear pain without barotrauma (*n* = 1), palpitations (*n* = 3), headache (*n* = 1) and fever (*n* = 1) [27]. These effects were generally manageable and did not impede the overall efficacy of the interventions. Conversely, rehabilitation and pharmacological treatment had no adverse effects.

### Brain-fog outcomes

#### Rehabilitation

figSix studies described the use of rehabilitation as a treatment modality [15, 18, 19, 22, 24, 25]. Each study employed a slightly different rehabilitation approach, resulting in varied outcomes regarding the success of the interventions. Table 4 illustrates the specific rehabilitation strategies implemented in each study. Four studies revealed enhancements in brain fog symptoms [19, 22, 24, 25], whereas two studies reported no significant improvement in brain fog symptoms [15, 18]. Kupferschmitt et al. performed a prospective cohort study of 80 post-COVID-19 patients who underwent multimodal rehabilitation. The multimodal rehabilitation concept included cognitive behavioral therapy and TAP, followed by cognitive training in a group setting (2 × 50 min/week) and in an individual setting (as needed), individualized aerobic exercise training, body awareness training, breathing therapy, relaxation techniques, and social counseling. The duration of rehabilitation was five weeks [15]. Kupferschmitt et al. indicated that depressive symptoms decreased to subclinical levels, as assessed by the Patient Health Questionnaire-9, both before admission and after discharge. However, cognitive deficits persist throughout the rehabilitation process, as measured by TAP scores [15]. Samper-Pardo et al. developed a mobile application named the ReCOVery app for their clinical trial involving 100 patients with long COVID-19. The intervention group utilized the mobile application in conjunction with the treatment-as-usual methods recommended by their general practitioners for three months [18]. The study's findings suggested that the use of the ReCOVery app for three months did not significantly enhance quality of life in patients with long-COVID [18]. In contrast to conventional rehabilitation methods, unconventional rehabilitation strategies have been explored in certain studies [18, 24, 25]. One such investigation by Hausswirth et al. involved a parallel randomized controlled trial in which 34 long-term COVID-19 patients were divided randomly into an intervention group (*n* = 17) and a control group (*n* = 17), with an additional 15 healthy individuals serving as a standard comparison group. The intervention cohort engaged in the Rebalance® Program, which emphasizes mindfulness-based interventions, encompassing ten sessions of 30 min each over four weeks [25]. Cognitive enhancements were gauged using the PEBL platform, revealing noticeable improvements in the intervention group, whereas the control group showed no significant cognitive enhancements. Moreover, the benefits of the mindfulness-based intervention appeared to persist, with cognitive improvements becoming more pronounced a week following the neuro-meditation intervention [25]. Additionally, Duñabeitia et al. studied 73 post-COVID-19 patients suffering from brain fog. Their objective was to mitigate these symptoms via personalized computerized cognitive training. Participants were assessed initially and after completing at least 10 training sessions across 8 weeks. The training regimen was customized to the individual cognitive profiles of the patients, as determined by the CAB, utilizing proprietary Individualized Training System software [24]. Posttraining, Duñabeitia et al. reported uniform improvements across various cognitive areas in posttest evaluations compared to the initial assessments, signifying enhancements following personalized computerized cognitive training [24].

**Table 4:** Rehabilitation approaches for each study that used rehabilitation as a treatment approach

Rehabilitation approach	Kupferschmitt et al. [15]	Samper-Pardo et al. [18]	Rabaiotti et al. [19]	Braga et al. [22]	Duñabeitia et al. [24]	Hausswirth et al. [25]	Wysokiński et al. [21]
Standard in-hospital rehabilitation program	v	-	v	v	-	-	-
Counseling	v	v	v	-	-	-	-
Group meetings	v	-	-	v	-	-	-
Test battery for attention	v	-	-	-	-	-	-
Cognitive training	v	v	v	-	v	-	v
Aerobic execrise training	v	-	v	-	-	-	-
Body awareness training	v	-	-	-	-	-	-
Breathing therapy	v	v	-	-	-	-	-
Telerehabilitation	-	v	-	-	-	-	-
Mindfullness	-	-	-	-	-	v	-

#### Noninvasive brain stimulation

Studies described the use of noninvasive brain stimulation as a treatment modality [14, 16, 21, 23, 26, 28]. Noninvasive brain stimulation involves a variety of techniques. In our review, we describe theta burst stimulation [14, 26], transcranial magnetic stimulation [16, 26], transcranial direct current stimulation [21], transcranial alternating current stimulation [23], and photobiomodulation [28]. Despite the use of various types of noninvasive brain stimulation, all studies have demonstrated improvements in brain fog symptoms [14].

#### Hyperbaric oxygen therapy

Three studies described the use of hyperbaric oxygen therapy as the treatment modality [20, 27, 29]. In all three studies, there was an improvement in perfusion (assessed by perfusion magnetic resonance imaging) and a reduction in brain fog symptoms.

#### Pharmacological

Two pharmacological studies met the inclusion criteria [17, 30]. De Luca et al. performed a clinical trial in which 69 long-term COVID-19 patients were divided into three groups: group 1, recurrent PEA-LUT plus olfactory training group (*n* = 43); group 2, recurrent pea-lut alone group (*n* = 16); and group 3, individuals who were exposed to olfactory training; these patients continued olfactory treatment while receiving PEA-LUT (*n* = 10). Cognitive impairment was assessed using the MMSE. Mental clouding showed a statistically significant reduction in severity between baseline and three months after treatment in groups 1 and 3. Group 2 showed no significant reduction in severity between baseline and three months after treatment [17]. Cenacchi et al. conducted an exploratory study comparing 26 patients treated with PEA-LUT to 15 who did not. They reported significant enhancements in the PRMQ and MoCA scores among those receiving PEA-LUT. Additionally, a secondary analysis of a subset of patients who received both PEA-LUT and corticosteroids (*n* = 7) versus those who received PEA-LUT alone (*n* = 19) revealed no significant difference in outcomes between the two groups [30].

### Secondary outcome

In addition to treating brain fog symptoms, each intervention managed to treat other comorbidities of the patients, more specifically long-COVID symptoms that are not brain fog.

#### Rehabilitation

One study showed that throughout rehabilitation depressive symptoms decreased to a subclinical level [15] through the assessment of the PHQ-9.

#### Noninvasive brain stimulation

The following improvements in symptoms were observed across various studies: neuropsychiatric manifestations (anxiety, depression) [14], chronic fatigue [16], insomnia [14], and visual field recovery [23]. Theta burst stimulation [14], low-frequency repetitive transcranial magnetic stimulation [16] and transcranial alternating current stimulation [23] were used.

#### Hyperbaric oxygen therapy

The following outcomes were observed across various studies: improvement in physical capacity (VO2 increased) and lung function (FVC, FEV, PEF) [20]; depression, anxiety, sleep, and pain interference symptoms [27]; and fatigue [20, 27, 29].

#### Pharmacological

Improvement in parosmia was observed alongside improvement in brain fog symptoms [17]. Table 5 summarizes the secondary outcomes and adverse effects for each intervention.

**Table 5:** Secondary outcomes and adverse effects for each intervention

Intervention	Secondary outcomes	Adverse effects
Noninvasive brain stimulation	Improvement of depression,anxiety, insomnia,fatigue and visual field(recovery)	Dizziness,headache,scalp pain at stimulation site
Rehabilitation	Depression	None
Hyperbaric oxygen therapy	Improvement of physical capicity(v02 increased), fatigue, lung functions(FVC,FEV,PEF), depression, anxiety, fatigue, sleep and pain interference symptoms	Barotrauma, ear pain, hypertension, palpitation, headache and fever
Pharmacological	Improved parsomia	None

Abbreviations: *FVC* Forced vital capacity; *FEV* Forced expiratory volume; *PEF* Peak flow measurement

## Discussion

This systematic review aimed to explore possible interventions for brain fog symptoms in long-COVID patients. Our review identified four main approaches: rehabilitation, noninvasive brain stimulation, hyperbaric oxygen therapy and PEA-LUT. While we did encounter several pharmacological studies during our search, they were not included in our analysis because they did not utilize formal cognitive assessment tools.

The rehabilitation approach exhibited the greatest variability among the three interventions. This diversity in rehabilitation methods has resulted in mixed outcomes across studies assessing this intervention approach. Two of the six studies [15, 18] demonstrated that rehabilitation did not yield improvements or that the cognitive assessment scores did not surpass those of the control group. There are several potential reasons why certain studies have failed to produce significant results. Both studies lacked personalized treatment; they implemented a multimodal approach in which all patients underwent the same interventions without considering individual symptoms or preferences. In contrast, studies focusing on personalized treatment have demonstrated improvements in cognitive assessment scores [19, 24]. Studies have shown that individualized rehabilitation approaches can lead to improvements in patients with cognitive impairment [31–33]. The study by Samper-Pardo et al. that used telerehabilitation proposed that the lack of results could be due to the participants not significantly using the mobile application or allowing it to be an effective tool. Pardo et al. reported that only 25% of the participants made significant use of the mobile application, indicating low adherence toward the mobile application. The mean age of the participants in the study by Samper-Pardo et al. was 48.28 years. Mizrachi et al. indicated that individuals above the age of 50 may encounter difficulties with the use of technology in the healthcare field [34]. Furthermore, in the study by Braga et al., out of the 208 patients enrolled, only 133 (63.9%) actively participated in the rehabilitation program by attending at least two out of the four scheduled meetings. A significant portion, 47 (22.6%), did not attend any meetings, while 28 (13.5%) attended only one. This pattern of low adherence to the rehabilitation program is consistent with findings from the study by Samper-Pardo et al. Braga et al. also noted that patients who did not engage in psychoeducational groups and reported no use of compensatory strategies had worse average total BNIS scores. These results underscore the importance of high adherence to the rehabilitation approach and offer another explanation for the mixed results. Regarding the improvement of brain fog symptoms in other rehabilitation programs, several studies support these findings. In a randomized controlled trial conducted by Nauta et al., individuals with multiple sclerosis and cognitive impairment underwent cognitive rehabilitation and mindfulness treatment. This study revealed improvements in mindfulness and cognitive rehabilitation. However, after six months of treatment, cognitive rehabilitation showed benefits only for personalized cognitive goals, while mindfulness demonstrated benefits only for processing speed [35]. A meta-analysis by Hill et al. regarding computerized cognitive training in older adults with mild cognitive impairment or dementia showed that computerized cognitive training was an effective treatment option for mild cognitive impairment [36].

Although noninvasive brain stimulation has been used in a variety of approaches, successful treatment approaches for brain fog symptoms have been identified. Currently, noninvasive brain stimulation treatment is primarily used in rehabilitation for conditions such as stroke, spinal cord injury, traumatic brain injury, and multiple sclerosis [37]. It has also shown efficacy in treating neuropsychiatric manifestations, particularly refractory depression [38]. The findings of improvement in brain-fog symptoms were supported by a meta-analysis conducted by Wang et al. This meta-analysis focused on noninvasive brain stimulation as an intervention for mild cognitive impairment and Alzheimer's disease. They found that noninvasive brain stimulation had a significant effect on global cognition, and the use of low-frequency repetitive transcranial magnetic stimulation over the dorsolateral prefrontal cortex improved memory function [39]. Noninvasive brain stimulation treatment primarily involves targeted neurostimulation of the dorsolateral prefrontal cortex and left orbitofrontal cortex. This approach was chosen because these regions are not only responsible for cognitive function but also because they can be directly and indirectly damaged by COVID-19 infection [40]. Noninvasive brain stimulation can facilitate stimulation of the dorsolateral prefrontal cortex, leading to improvements in neural rhythms, including theta and gamma amplitude coupling, which is also related to cognitive function and may even lead to enhanced neuroplasticity [41, 42]. The theory behind the success of these treatments is complex and not completely understood; however, one such example is cortical excitability and neuroplasticity. Cortical excitability refers to the intrinsic responsiveness of neurons in the cerebral cortex to excitatory and inhibitory inputs, which can be modulated by noninvasive brain stimulation [43]. These techniques induce changes in neuronal membrane potentials, synaptic efficacy, and neurotransmitter release, consequently altering the excitability of targeted brain regions. Concurrently, noninvasive brain stimulation treatments also facilitate neuroplasticity, the brain's ability to reorganize its structure and function in response to external stimuli or experiences. Neuroplastic changes induced by noninvasive brain stimulation involve synaptic remodeling, dendritic growth, and alterations in neural network connectivity, contributing to the adaptive responses observed following stimulation. The interplay between cortical excitability and neuroplasticity underlies the efficacy of noninvasive brain stimulation treatments across various neurological and neuropsychiatric conditions [44]. By enhancing cortical excitability and promoting neuroplasticity, noninvasive brain stimulation interventions harness the inherent plasticity mechanisms of the brain to promote recovery, alleviate symptoms, and improve cognitive function in affected individuals.

Hyperbaric oxygen therapy involves the delivery of 100% oxygen at environmental pressures exceeding one atmosphere. This process significantly increases the partial pressure of oxygen in the blood and tissues beyond what is achievable with standard oxygen supplementation [45]. Currently, it is acknowledged as an effective treatment method for a range of brain injuries [46, 47]. A study conducted by Chen et al. recruited patients with Alzheimer's disease and amnestic mild cognitive impairment for hyperbaric oxygen therapy, consisting of 40 min of treatment daily for 20 days. The results of the study showed that hyperbaric oxygen therapy significantly improved cognitive function, as assessed by the MMSE and MoCA [48]. This finding is consistent with the results of the studies included in our review. Hyperbaric oxygen therapy is an effective treatment for brain fog symptoms for several reasons. The common reason is the improvement in tissue oxygenation; moreover, improvement in tissue oxygenation was suggested to be a supportive therapy for COVID-19 [49]. Studies have indicated that hyperbaric oxygen therapy not only increases tissue oxygenation but also affects oxygen and pressure-sensitive genes, thereby promoting regenerative processes, such as stem cell proliferation and neurogenesis [50, 51]. This suggests that hyperbaric oxygen therapy may induce neuroplasticity and subsequently improve cognitive function [52–54].

Both studies investigating PEA-LUT reported positive outcomes for the use of this drug. The primary rationale for the effectiveness of PEA-LUT is attributed to its anti-inflammatory and neuroprotective effects, positioning it as an antagonist of neuroinflammation [55]. PEA, an innate component of the N-acylethanolamine family found in numerous tissues, including the brain, is synthesized in response to stress to restore tissue equilibrium. The success of PEA in ameliorating cognitive deficits may be due to its ability to inhibit the nuclear factor-κB (NF-κB) pathway by activating PPAR-α receptors [56], thereby dampening brain inflammation. This action is vital for addressing cognitive dysfunctions such as memory issues and brain fog. PEA’s neuroprotection potentially shields neurons against inflammatory harm, which is pertinent for long-COVID neurological symptoms [57]. Furthermore, PEA augments anandamide function—a neurotransmitter that modulates pain, appetite, and memory—by interacting with the cannabinoid-like receptors GPR55 and GPR119 [58], possibly restoring compromised neurotransmission in long-COVID cognitive disorders. By reducing oxidative stress and modifying inflammatory pathways, PEA may contribute to the alleviation of cognitive symptoms such as brain fog, underscoring its therapeutic promise for symptom management [59].

**Fig. 3:**
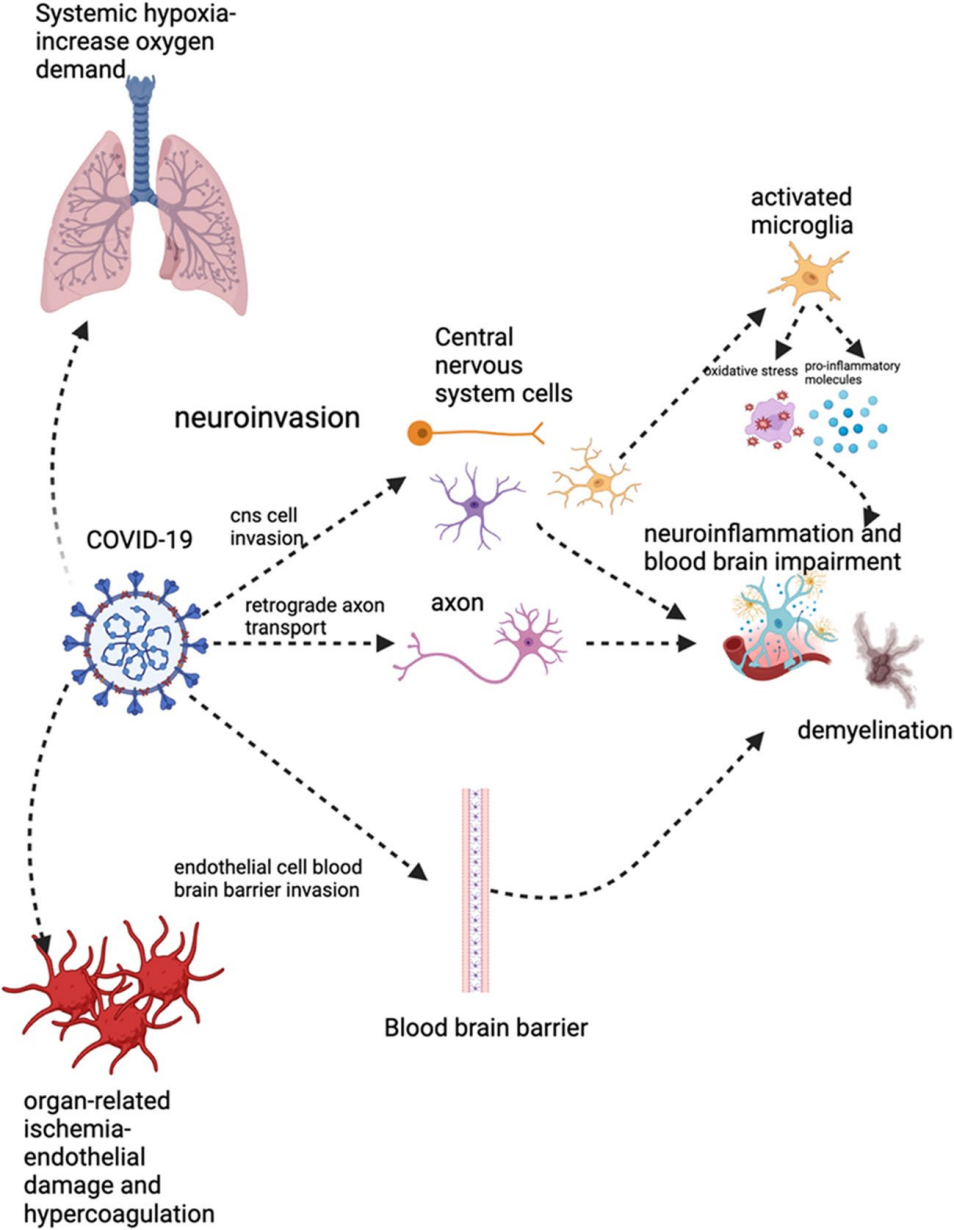
Potential mechanism underlying brain-fog symptoms caused by COVID-19. When COVID-19 patients gain access to the central nervous system (CNS), various pathways are activated, including direct invasion of CNS cells, retrograde axonal transport, and penetration through the endothelial cells of the blood‒brain barrier. Once inside the CNS, COVID-19 can prompt microglia to release proinflammatory agents, leading to mitochondrial dysfunction and oxidative stress. This cascade of events can lead to neuroinflammation, demyelination, and neurodegeneration. Moreover, systemic hypoxia and organ-related ischemia also participate in pathogenesis

The mechanism behind brain fog symptoms post-COVID-19 is not completely understood. There are a variety of hypotheses. One prevailing hypothesis suggests that COVID-19 infects cells in the central nervous system through ACE2 receptors, particularly astrocytes, which are abundant in the central nervous system. When infected, astrocytes may modify their metabolic pathways, potentially causing harm to neighboring neurons, as astrocytes provide support to neurons. This disruption may explain the symptoms observed in individuals experiencing brain fog [60]. Another theory suggests that microglia are activated, possibly triggered by an entry point from the hypothalamus. The activation of these microglia can lead to the release of proinflammatory molecules [61]. Additionally, COVID-19 may worsen oxidative stress and cause mitochondrial dysfunction in microglia [62]. These neuroinflammatory responses and impaired redox processes are believed to be significant factors in the progression of neurological effects associated with prolonged COVID-19 [63]. In addition to inflammation, hypoxia plays a significant role in the pathogenesis of post-COVID-19 conditions. Systemic hypoxia arises from lung impairment, and patients with persistent lung issues often require supplemental oxygen. Studies have shown a correlation between cognitive impairment and the degree of oxygen supplementation required to alleviate respiratory challenges [64]. Furthermore, COVID-19 can induce organ-related ischemia by causing endothelial damage and hypercoagulation, thereby increasing the risk of vascular dysfunction [65]. The potential mechanisms underlying brain fog symptoms are shown in Fig. 3.

Based on our review, the possible interventions for brain fog include rehabilitation, noninvasive brain stimulation, and hyperbaric oxygen therapy. However, determining the most suitable intervention for individual patients is challenging. Due to the use of different outcome measures in the studies, we could not determine which intervention showed the most success regarding the primary outcome, treating brain fog symptoms. Nevertheless, certain parameters, such as treatment duration, adverse effects, and secondary outcomes, should be considered when considering which intervention to use. Patients who prefer shorter intervention methods may benefit from noninvasive brain stimulation, which has shown the lowest number of sessions required and the shortest time to treat brain fog symptoms. Moreover, patients with neuropsychiatric manifestations will benefit because noninvasive brain stimulation is useful for treating neuropsychiatric symptoms such as depression. Hyperbaric oxygen therapy, despite requiring a greater number of sessions, offers benefits in improving tissue perfusion, making it beneficial for patients experiencing chronic fatigue due to long-term COVID-19 and for patients suffering from persistent lung function impairment. Rehabilitation appears to be the safest choice among the interventions, with no reported adverse effects, and a study suggested possible improvements in depression symptoms. However, further research is necessary to ascertain the efficacy of rehabilitation due to the mixed results observed. An important aspect to consider is the significance of adherence to the treatment regimen for achieving successful outcomes. Therefore, rehabilitation may be recommended for patients with high adherence who prefer interventions without potential adverse effects or who are apprehensive about noninvasive brain stimulation and hyperbaric chamber treatments. Due to the limited number of studies on pharmacological interventions, it is challenging to determine their effect on the treatment of brain fog. However, based on the available studies, PEA-LUT is a possible treatment for brain fog and has been shown to be effective in treating parosmia. Further research is needed to determine the optimal pharmacological intervention for brain fog symptoms in post-COVID-19 patients (Fig. 3).

It is important to note that only one study combined rehabilitation with other interventions. Wysokiński et al. combined transcranial direct current stimulation with cognitive rehabilitation. They concluded that transcranial direct current stimulation combined with another therapeutic intervention (caused by suprathreshold stimuli) may serve as an inducer of neuroplasticity, thus amplifying the training effects of cognitive rehabilitation. This hypothesis is supported by a study by Rodella et al., who combined transcranial direct current stimulation with cognitive training in patients with mild cognitive impairment [66]. Further studies comparing the efficacy of transcranial direct current stimulation alone versus transcranial direct current stimulation combined with cognitive training are necessary to better understand their respective impacts on brain fog symptoms and cognitive function.

The strength of our study was that we performed a comprehensive search of a wide number of electronic databases. The limitations of this review are the limited number of studies included, high heterogeneity due to the use of different scoring methods, the inclusion of case reports and case series with very small sample sizes, and the lack of meta-regression analysis.

## Conclusion

The importance of finding the right intervention for brain fog symptoms is an important task for physicians because of the reduced quality of life and difficulty returning to their normal occupations. Our review revealed that noninvasive brain stimulation and hyperbaric oxygen therapy show promising results in the treatment of brain fog symptoms caused by long-COVID, showcasing improved perfusion and cortical excitability. Furthermore, both rehabilitation strategies and PEA-LUT administration have been associated with improvements in symptoms of brain fog. Future studies should explore combinations of interventions and include longer follow-up periods to assess the long-term effects of these treatments.
